# Point-of-care ultrasound: The new district focus

**DOI:** 10.4102/phcfm.v15i1.3576

**Published:** 2023-06-14

**Authors:** Louw Fourie, Michael K. Pather, Gavin Hendricks

**Affiliations:** 1 Division of Family Medicine and Emergency Medicine, Faculty of Medicine and Health Sciences, Stellenbosch University, Cape Town, South Africa

**Keywords:** point-of-care ultrasound, district hospitals, under-resourced settings, curriculum, West Coast District

## Abstract

**Background:**

Point-of-care ultrasound (POCUS) improves patient outcomes. The current POCUS curriculum of the Emergency Medicine Society of South Africa is based on guidelines from the United Kingdom with a different burden of disease (BoD) and available resources than encountered locally.

**Aim:**

To determine which modules of the POCUS curriculum should be implemented to better equip doctors working at a district hospital in the West Coast District (WCD), South Africa.

**Setting:**

Six district hospitals within the WCD.

**Methods:**

A descriptive cross-sectional survey with questionnaires for medical managers (MMs) and medical practitioners (MPs).

**Results:**

A response rate of 78.9% for MPs and 100% for MMs was obtained. MPs rated the following modules of POCUS most relevant to their daily practice: (1) first trimester pregnancy; (2) deep vein thrombosis; (3) extended focused assessment with sonography in trauma; (4) central vascular access; and (5) focused assessment with sonography for human immunodeficiency virus (HIV) and tuberculosis (TB) (FASH).

**Conclusion:**

There is a need for a POCUS curriculum informed by the local pattern of disease. Priority modules were identified based on the local BoD and reported relevance to practice. Despite the availability of ultrasound machines within the WCD, few MPs were accredited and able to perform POCUS independently. There is a need to implement training programmes for medical interns, MPs, family medicine registrars and family physicians working in district hospitals. A relevant curriculum for POCUS training based on the local needs within communities has to be developed.

**Contribution:**

This study emphasises the need for a locally informed POCUS curriculum and training programmes.

## Introduction

The use of point-of-care ultrasound (POCUS) has significantly increased over the past several years and has become the standard of care in the disciplines of emergency medicine, anaesthesiology and critical care units.^1^ Point-of-care ultrasound surpasses conventional ultrasound in speed, accessibility and availability by bringing the ultrasound to the patient as opposed to the conventional pathway of referring the patient to a specialist or radiology department.^2^ Although POCUS originated in emergency medicine, literature supports the movement of its application to other specialties including family medicine and primary health care (PHC), which include non-specialist medical practitioners (MPs) as a safe and accurate imaging application.^3,4,5,6,7,8,9^ Assessment of general practitioners showed that focused scans require less time to perform and less training, have higher levels of accuracy and are also associated with less potential for harm than more comprehensive scans.^10^ The ability of MPs to use POCUS at the patient’s bedside has improved patient care significantly with decreased time to diagnosis and length of stay in the emergency unit, rapid assessment of critically ill patients and higher success rates of procedures using ultrasound guidance.^11,12,13,14^

There is currently no POCUS curriculum for generalist doctors in family medicine and district hospitals. The Emergency Medicine Society of South Africa (EMSSA) offers emergency point-of-care ultrasound (ePOCUS) training for its registrars and other interested doctors.^9^ The core ultrasound modules in ePOCUS were: (1) extended focused assessment with sonography in trauma (eFAST), (2) abdominal aorta aneurysm (AAA) diagnosis, (3) central and peripheral vascular access, (4) deep vein thrombosis (DVT) diagnosis and (5) focused emergency echocardiography in resuscitation (FEER).^15,16,17^ The ePOCUS curriculum was based on guidelines from the United Kingdom, although this is not ideal given the differences between the healthcare systems, disease burden and context of the two countries.^16,17^ A South African study found that the transferred curriculum did not address the local burden of disease (BoD) and little consideration was taken for the lack of staff and technological resources.^16^ Another study found that eFAST, DVT, FEER, first trimester pregnancy and focused assessment with sonography for human immunodeficiency virus (HIV) associated extra-pulmonary tuberculosis (TB) (FASH) were the modules that emergency MPs most frequently used in their daily clinical work.^17^ New 2021 guidelines by EMSSA make a distinct difference between core (image acquisition and optimisation; eFAST; focused AAA; basic cardiac ultrasound; basic lung ultrasound; ultrasound-guided vascular access) and more advanced POCUS modules (mandatory: focused cardiac ultrasound and haemodynamic assessment; DVT extended compression ultrasound; advanced thoracic (lung) and airway ultrasound; optional: regional anaesthesia and nerve blocks; hepatobiliary and genito-urinary tract POCUS; gastrointestinal tract POCUS (including FASH); focused obstetric and gynaecological POCUS; transcranial Doppler and ocular ultrasound), which could be individualised.^9^ It still maintains its international focus that is stipulated by the International Federation for Emergency Medicine (IFEM).^18^

Low- to middle-income countries are faced with healthcare challenges including a lack of specialised healthcare force and limited diagnostic infrastructure.^19^ Determining the local disease burden and needs assessment will allow conception of context adapted POCUS modules and education programmes.^19^ To better understand the curricular needs within the district public healthcare system, we must first look at the BoD. South Africa faces a quadruple BoD as evidenced by years of life lost in 2017 caused by: non-communicable disease (45.6%), HIV and TB (22.3%), communicable disease (includes maternal, perinatal and nutritional disorders) (16.9%) and injuries (15.2%).^20^ The five leading causes of premature mortality (% contribution in brackets) in South Africa are: (1) HIV and acquired immunodeficiency syndrome (AIDS) (12.7%), (2) TB (9.6%), (3) lower respiratory tract infections (6.7%), (4) cerebrovascular accidents (5.1%) and (5) diabetes mellitus (4.1%).^20^

In South Africa, emergency medicine is the only specialist field that currently offers POCUS training that could be used by doctors in the district health system. It is deemed a requirement by the College of Emergency Medicine of South Africa and also endorsed by EMSSA.^9^ The expertise for training candidates in POCUS exists, but what else is required to implement a successful curriculum? As identified here, disease burden is a major contributing factor as well as the technical difficulty of generating and interpreting quality ultrasound images to make accurate diagnoses.^9,17^ Despite the strategies that could be applied to successfully implement a context-relevant POCUS curriculum, considering the aforementioned factors, there are still many barriers that would need to be overcome. Examples of barriers from similar healthcare settings are: (1) poor access to reliable power; (2) stock outs (e.g. ultrasound jelly); (3) limited access to ultrasound machines; (4) a lack of portable ultrasound machines; (5) limited access to trainers (credentialed); (6) time constraints for training and a lack of support (National, provincial and health authorities).^15,17,21^

Point-of-care ultrasound holds many advantages over conventional imaging modalities. It is portable, non-invasive, reproducible, carries no ionising radiation risk and is less expensive than other imaging modalities (x-rays; computed tomography and magnetic resonance imaging).^14^ Basic lung POCUS is a more reliable and faster modality to identify lung pathology (haemo-, pneumo-thorax; pleural effusion; pulmonary oedema; pneumonia) than x-rays.^22,23^ South Africa is a resource-limited country; therefore, POCUS would be an ideal imaging modality, especially in a district or rural setting. There are many benefits that POCUS holds for the patient and clinician, which include supporting and excluding diagnoses; directing treatment that should be instituted; reducing radiology expenses and supporting referral of patients to appropriate centres for definitive treatment.^1,9,14,24,25^

When considering recommendations for modules that will eventually be applied in medical curricula, evidence-based methods should be used to identify modules in medical education.^26^ Appropriate training programmes ensure operator competence and patient safety.^9^ In North America, medical schools are integrating POCUS into their curricula with protocols for family physicians performing ultrasounds (family medicine ultrasound [FAMUS]).^27,28^

Current applications for POCUS at a district level in South Africa have not been evaluated yet. Modules are based on that of the international community and were similar to that of the United Kingdom curriculum.^17,18^ The direct transfer of curriculum from first-world countries is however not recommended as the need and BoD may differ in resource-limited countries such as South Africa.^15,16,29,30^ To ensure the correct utilisation of resources and to address the local BoD, relevant modules of POCUS need to be included in a needs-based curriculum. This research aims to fill the gap between the current curriculum implemented by the EMMSA in South Africa and the modules required for district healthcare settings. Addressing the specific need ensures less wasted expenditure, focused and efficient training to healthcare providers, improved healthcare services to the community and improved outcomes for patients.

### Aim

This study aimed to determine which modules of the POCUS curriculum should be implemented in a training programme for doctors working at a district hospital in the West Coast District (WCD), Western Cape, South Africa.

## Research methods and design

### Study design

This was a descriptive cross-sectional study. A survey with two questionnaires was used, one for medical managers (MMs) and one for MPs.

### Study setting

The survey was conducted within the WCD of South Africa. The district consists of five sub-districts, which include: (1) Swartland, (2) Bergrivier, (3) Matzikama, (4) Cederberg and (5) Saldanha Bay. The total population for this area is estimated at 464 056.^31^ Permission was granted to conduct the study from the Western Cape Department of Health (DoH), applied through the National Health Research Database (NHRD), reference number WC202101_008. Each MM at the six district hospitals within the region (Citrusdal, Clanwilliam, Swartland [Malmesbury], Radie Kotze [Piketberg], Vredenburg and Vredendal) also granted permission for the study to be conducted at their respective facilities.

### Study population

Each MM at each district hospital, as well as all the MPs (57)employed therein, were invited to participate in the study survey. Interns were excluded as they work under supervision.

### Questionnaire

Two questionnaires were designed, one for MPs and one for MMs. The questionnaires were piloted with the research supervisors to ensure that the instructions and questions were clear and logical. An electronic consent form preceded the questionnaires.^32^ Research Electronic Data Capture (REDCap) software was used to distribute the questionnaires online. Coronavirus disease 2019 (COVID-19) measures were taken into account as not to have any physical meetings with MMs or MPs.

Demographic data were collected for both MMs and MPs. Regarding MMs, the focus was on imaging facilities that were available and who provided ultrasound at their facilities. For MPs, the question focus was on their training and accreditation and ability to perform POCUS independently, and a Likert scale that rated the relevance of each module of POCUS to their daily practice.

### Data collection

Data were captured over a 2-month period. Two online questionnaires, one for MPs and one for MMs were used. Study data were collected and managed using REDCap tools hosted at Stellenbosch University.^33,34^ REDCap is a secure, web-based software platform designed to support data capture for research studies, providing: (1) an intuitive interface for validated data capture; (2) audit trails for tracking data manipulation and export procedures; (3) automated export procedures for seamless data downloads to common statistical packages and (4) procedures for data integration and interoperability with external sources.^33,34^

All communication took place telephonically and via email. Medical managers were contacted telephonically to ensure the participation of their respective facilities (including MPs who are employed at their facility). Online questionnaires (one for MPs and one for MMs) were sent out using the REDCap platform. Data were collected anonymously to ensure confidentiality and reduce bias.

### Data analysis

Data analysis was performed at Stellenbosch University by exporting the collected data to the Statistical Package for Social sciences (SPSS) version 25.^35^ Data were reviewed and cleaned before analysis was performed. Descriptive statistics were explored and described using the median and interquartile range (IQR) as the data were not normally distributed. The Pearson Chi-squared test and Fisher–Freeman–Halton Exact test were used to describe categorical and binary data points. The Mann–Whitney *U* test was used to describe non-normally distributed data points. Tables and graphs were constructed in Microsoft Excel 2016.

### Ethical considerations

Ethics approval was obtained from Stellenbosch University Health Research Ethics Committee S20/07/159. Consent was obtained electronically from all participants and they were free to withdraw from the study at any time. Participants completed the questionnaires anonymously and data were kept confidential.

## Results

A total of 63 participants, 57 MPs and 6 MMs, were invited to participate in this research study. Of these, 51 participants completed their questionnaires (45 MPs and 6 MMs). There were no missing data in any of the completed questionnaires. Overall, the response rate was 78.9% and 100% for MPs and MMs, respectively.

The median age of MPs was 29 years [IQR: 6.5] with a female, 29 (64.4%), to male, 16 (35.6%), predominance. The median number of years practising post-internship was 3 [IQR: 5.5]. The majority of the group consisted of medical officers, 29 (64.4%), followed by community service medical officers, 13 (28.9%), registrars, 2 (4.4%) and one (2.2%) specialist. All hospitals described in the study setting were represented. The largest proportion of participants was from Swartland Hospital, 18 (40%), and Citrusdal Hospital comprised the smallest number of MPs, 2 (4.4%). The mean age for MMs was 51.7 years and one MM from each of the six facilities completed a questionnaire.

A minority of MPs reported that they were trained and accredited in POCUS (eFAST 4 [8.9%], DVT 2 [4.4%], FEER 3 [6.7%], central vascular access 6 [13.3%], AAA 2 [4.4%] and FASH 1 [2.2%]). For first-trimester pregnancy, 17 (37.8%) MPs indicated that they were trained and accredited to perform this element (Table 1). Only a minority of respondents indicated that they were able to perform elements of POCUS independently (eFAST 7 [15.6%], DVT 4 [8.9%], FEER 4 [8.9%], central vascular access 9 [20%], AAA 6 [13.3%] and FASH 2 [4.4%]). For first-trimester pregnancy, 34 (75.6%) reported that they were able to perform this independently (Table 2).

**TABLE 1:** Medical practitioners trained and accredited.

POCUS element	*N*	%
**eFAST**		
No	41	91.1
Yes	4	8.9
**DVT**		
No	43	95.6
Yes	2	4.4
**FEER**		
No	42	93.3
Yes	3	6.7
**Central vascular access**		
No	39	86.7
Yes	6	13.3
**AAA**		
No	43	95.6
Yes	2	4.4
**FASH**		
No	44	97.8
Yes	1	2.2
**First-trimester pregnancy**		
No	28	62.2
Yes	17	37.8

POCUS, point-of-care ultrasound; DVT, deep vein thrombosis; eFAST, extended focused assessment with sonography in trauma; FEER, focused emergency echocardiography in resuscitation; AAA, abdominal aorta aneurysm; FASH, focused assessment with sonography for HIV/TB.

**TABLE 2:** Medical practitioners’ ability to perform point-of-care ultrasound independently.

POCUS element	N	%
**eFAST**		
No	38	84.4
Yes	7	15.6
**DVT**		
No	41	91.1
Yes	4	8.9
**FEER**		
No	41	91.1
Yes	4	8.9
**Central vascular access**		
No	36	80.0
Yes	9	20.0
**AAA**		
No	39	86.7
Yes	6	13.3
**FASH**		
No	43	95.6
Yes	2	4.4
**First-trimester pregnancy**		
No	11	24.4
Yes	34	75.6

POCUS, point-of-care ultrasound; DVT, deep vein thrombosis; eFAST, extended focused assessment with sonography in trauma; FEER, focused emergency echocardiography in resuscitation; AAA, abdominal aorta aneurysm; FASH, focused assessment with sonography for HIV/TB.

All six facilities offered x-ray imaging daily from 8:00 to 16:00. Only four facilities offered limited after-hours x-ray services. There were a total of 10 functioning ultrasound machines in the WCD at the time of the survey. Only Vredendal Hospital reported that they offered ultrasound services 5 days per week, performed by an accredited sonographer. Two hospitals reported that they offered after-hours ultrasound services (Citrusdal and Clanwilliam). Swartland Hospital also had a sonographer and radiographer, whereas Vredendal Hospital only had a sonographer to perform ultrasound services.

**FIGURE 1:**
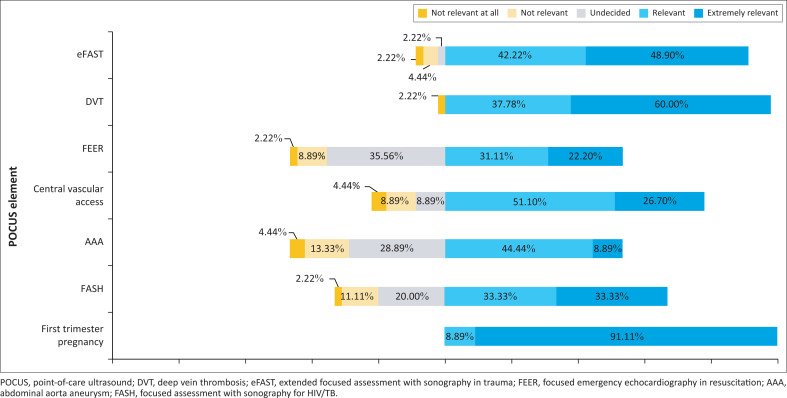
Point-of-care ultrasound relevance in daily practice.

Five hospitals indicated that MPs performed POCUS. A total of 34 (75.6%) MPs reported that POCUS was performed daily at the hospitals. None of the facilities had a radiologist who carried out ultrasound services at any of the facilities. A total of 44 (97.8%) MPs indicated that they were interested to train in POCUS. A Likert scale was used to rate the relevance of POCUS modules to MPs daily practice. The results of the Likert scale were visually represented by a stacked bar graph to illustrate the relative position of modules to each other. The modules were ranked in order of importance and the first-trimester pregnancy POCUS, DVT and eFAST were identified as the top three (Figure 1).

The Pearson Chi-squared test was used to compare the relationship between MPs’ level of employment and their ability to perform POCUS. Fisher–Freeman–Halton Exact test was also incorporated to control for a small study sample. A statistically significant relationship between the ability to perform first-trimester pregnancy POCUS and the level of employment was found (Pearson Chi-squared test: 12 994; *p*-value: 0.003; Fisher–Freeman–Halton Exact test: 11.514; *p*-value: 0.003). We also compared the level of employment with being trained and accredited in POCUS. A statistically significant relationship was found between being trained and accredited in first-trimester POCUS and the level of employment (Pearson Chi-squared test: 8.140; *p*-value: 0.020; Fisher–Freeman–Halton Exact test: 8.620; *p*-value: 0.013).

A Mann–Whitney *U* test revealed that there was a statistically significant association between the years post-internship and the ability to perform the following modules of POCUS: eFAST *U* = 201.0, *p* = 0.030; DVT *U* = 141.0, *p* = 0.020 and AAA *U* = 209.0, *p* = 0.020. No correlation was shown for FEER, central vascular access, FASH or first-trimester pregnancy POCUS. A comparison was also made between the number of years post-internship and being trained and accredited in POCUS. The Mann–Whitney *U* test revealed that there was a statistically significant association between this variable and the following elements of POCUS: FASH *U* = 45, *p* = 0.044; first-trimester pregnancy *U* = 355.0, *p* = 0.006. There was no correlation for any of the other elements.

## Discussion

The BoD in South Africa, the Western Cape province and the WCD, all differ. The core modules offered by the EMSSA (eFAST; focused abdominal aortic ultrasound (US); basic cardiac ultrasound; basic lung ultrasound; ultrasound-guided vascular access)^9^ do not correspond well with the BoD in South Africa, as described in the District Health Barometer 2019/20.^36^ There is however a better correspondence for the Western Cape province and the WCD as three of the top five conditions described in the BoD correspond to core POCUS modules (basic lung US; basic cardiac US; eFast; vascular access; focused abdominal aortic US). Advanced elements of POCUS (e.g. gastrointestinal-tract POCUS, which includes FASH and focused obstetric and gynaecological POCUS, which includes first-trimester pregnancy) are also needed, in addition to core modules, to manage patients effectively in these settings.

The types of POCUS practised in a setting will largely depend on the BoD and emergencies that occur in that setting.^9^ However, disease impact on the system and technical difficulty to acquire images should also be considered.^17^ It is, therefore, crucial to have a curriculum that is based on the local need and BoD.^9,16,37^ This is to equip and empower MPs to better identify and manage emergency conditions in their district.^16^ From the BoD in the WCD, we can see that healthcare providers, who are trained and accredited in POCUS, as well as their patients, would benefit from an area-specific training curriculum.

Based on the BoD and results on how MPs rated the relevance of POCUS elements to their daily practice, priority POCUS modules in the WCD would be: (1) basic lung US, (2) basic cardiac US, (3) eFAST, (4) first-trimester pregnancy (focused obstetric and gynaecological POCUS), (5) DVT (extended compression ultrasound) and (6) gastrointestinal-tract POCUS (including the FASH protocol). To prioritise these modules would assist MPs to be better equipped to manage scenarios that they frequently encounter. Internationally, general practitioners are often the first responders in various medical situations. In Canada, Norway, Australia and New Zealand, many general practitioners often work in rural areas too.^38,39,40^ They form a vital link in medical and emergency care for initial stabilisation, diagnosis and management of critically ill patients.^41,42,43^ Possessing POCUS skills for MPs could hold great potential benefit for themselves and patient care.^41^ Thus, to develop a POCUS curriculum for MPs who work in rural areas, like the WCD, would prove to be beneficial.

Researchers established that a minority of MPs in the WCD were trained and accredited in POCUS as well as had the ability to independently perform elements of POCUS daily. This finding is supported by a recent study performed in KwaZulu-Natal, South Africa, which found that only 7% of POCUS examinations carried out in the emergency department, were performed by accredited level 1 emergency POCUS providers.^44^ The reason for this could be that further training in POCUS is needed to become a provider and be accredited to ensure consistent, reproducible, and quality examinations and interpretation. Limited specialties offer POCUS training in their curriculum (i.e. emergency medicine – Western Cape faculty, Eastern-Cape faculty; KwaZulu-Natal faculty and Gauteng faculty [Johannesburg and Pretoria]).^9,15^ However, EMSSA offers training courses for non-emergency medicine candidates. Companies that offer training in POCUS in line with EMSSA guidelines would also have their support.^9^ EMSSA remains the only credentialing organisation.^9^

There was a large number of medical MPs in this study who reported that they had the ability to perform POCUS for the first-trimester pregnancy. In South Africa, all pregnant women should have at least one ultrasound.^45^ Due to resource limitations, this may be performed by radiographers, midwives (basic ultrasound course), medical officers and family physicians in addition to the preferred accredited ultrasonographers.^45^ Doctors may learn valuable skills in their undergraduate years in obstetrics and gynaecology to perform basic ultrasound in pregnancy. They may continue developing this skill while working as interns, community service doctors and medical officers on a vocational platform. This could explain why 75.6% of respondents reported that they were able to perform ultrasound for first-trimester pregnancy independently.

The researchers found that all six facilities lacked formal ultrasound services. These facilities thus had to rely on their MPs to perform POCUS on days that they did not have a formal service. Evidence suggests that POCUS can be more accurate than conventional cardiac, respiratory examination and x-ray imaging.^46,47^ National, provincial and district-level figures on the BoD 2019/2021 show a high BoD regarding respiratory disease and trauma.^36^ When we consider the BoD in the WCD, we see the valuable role that POCUS can play in the diagnosis and management of respiratory and cardiac conditions (pneumonia, pleural effusions, pneumothoraxes, work-up of pulmonary oedema and dyspnoea) as well as trauma (haemo-, pneumo-thorax; haemoperitoneum; haemopericardium) and haemodynamically unstable patients (ruptured ectopic pregnancy or aortic aneurysm).^9, 48,49,50,51^

Enabling doctors to learn and practise POCUS from an undergraduate level may thus be beneficial to the whole healthcare system in South Africa. This is especially relevant while considering the BoD in context. The POCUS training has been utilised in medical schools in the United States and Canada to enhance student’s medical education.^52^ Undergraduate students and interns in South Africa are not offered any formal POCUS training.^9^ This leaves them unable to perform and apply POCUS when they commence their medical careers as community service officers and onwards.^53^ This was also reflected in our results where we found that there was a statistically significant relationship between the years post-internship and the ability to perform POCUS. This relationship is indicative of a need for the inclusion of POCUS curricula in early career training. The EMSSA is open to other specialties and doctors, apart from those in emergency medicine, to train in POCUS.^54,55,56^ Specialties would be encouraged to develop their own guidelines as per their needs, still based on and in collaboration with those of emergency medicine.^9^

### Future recommendations

A clearer understanding is required of where and how MPs were trained and accredited as re-credentialing is necessary every fourth year.^9^ This study identified priority POCUS modules. The importance of POCUS modules within the specific districts and provinces should also be determined and ranked according to priority.^16^ Establishing a curriculum that is commenced at an undergraduate level and continued in internship will be a large endeavour that needs to be supported by the DoH, the Health Professions Counsel of South Africa (HPCSA) and the national universities.^9^ Engaging in discussion with the relevant stakeholders, one could further motivate for a change in practice. Medical practitioners form the backbone of the workforce on district health platforms. Family medicine registrars and family physicians are fundamentally involved with the district and PHC system.^57^ Including them in training programmes could prove beneficial especially in poorly resourced settings. Future applications for POCUS in the family medicine curriculum could also be explored. Possible barriers in POCUS training need to be identified and addressed to ensure adequate access to accredited programmes. The current data also need to be adjusted to the post-COVID-19 world, where health needs and priorities may have changed.

### Limitations

Some of the survey questions were ambiguous. Question six for MPs could have been misinterpreted as it was double-barrelled for being trained and accredited in elements of POCUS. The relevance of POCUS modules to daily practice, ranked by participants, were subjective opinions as not all participants were trained and accredited.

## Conclusion

The aim of this cross-sectional study was to determine which modules of the POCUS curriculum could be implemented to better equip doctors working at a district hospital in the WCD. This study highlights the need for a POCUS curriculum that addresses the local BoD in the WCD. The results indicated that current POCUS skills within the district are lacking. Despite the availability of ultrasound machines within the WCD, few MPs were accredited and able to perform POCUS independently. Priority modules were identified based on the local BoD and the reported relevance to daily practice. These were basic lung US, basic cardiac US, eFAST, the first-trimester pregnancy (focused obstetric and gynaecological POCUS), DVT (extended compression ultrasound) and gastrointestinal-tract POCUS (including the FASH protocol). There is a need to implement training programmes for medical interns, MPs, family medicine registrars and family physicians working in district hospitals. The findings from this study open up the conversation to develop a relevant curriculum for POCUS training based on the local needs within communities.
